# Longitudinal Perspectives on Depressive Symptoms Among Older Adults or Their Caregivers in Asia

**DOI:** 10.1093/geroni/igaf122.213

**Published:** 2025-12-31

**Authors:** Jeremy Lim-Soh, Rahul Malhotra

## Abstract

Mental health is critical for older adults and their caregivers, both as a contributing factor to health behaviors and other health outcomes, and an important outcome in its own right. This symposium advances our understanding of older adults’ or their caregivers’ mental health, specifically depressive symptoms, in three ways. First, it focuses on studies that harness longitudinal data to provide a more robust and comprehensive picture of how depressive symptoms change over time, identify causal factors for depressive symptoms and the impact of depressive symptoms on healthcare use. Second, it investigates depressive symptoms in the context of aging in Asia, where expectations of support from kin and non-kin are different from some Western contexts, leading to potentially contrasting outcomes for mental health when such expectations are not met. Third, it explores various facets of social support, including social networks, family caregiving, and elder abuse, and their implications for depressive symptoms. Together, the findings call for earlier identification and management of depressive symptoms in older adults or their caregivers, and their risk factors. While social support can help to mitigate depressive symptoms, policymakers and practitioners should be sensitive to the complex and context-dependent relationship between support and depressive symptoms for older adults and their caregivers.

